# Wear Performance Analysis of Ni–Al_2_O_3_ Nanocomposite Coatings under Nonconventional Lubrication

**DOI:** 10.3390/ma12010036

**Published:** 2018-12-22

**Authors:** Muhammad Usman Bhutta, Zulfiqar Ahmad Khan, Nigel Garland

**Affiliations:** 1 Department of Design & Engineering, NanoCorr, Energy & Modelling (NCEM) Research Group, Talbot Campus, Bournemouth University, Fern Barrow, Poole BH12 5BB, UK; zkhan@bournemouth.ac.uk (Z.A.K.); ngarland@bournemouth.ac.uk (N.G.); 2 School of Mechanical & Manufacturing Engineering (SMME), Campus H-12, National University of Sciences & Technology (NUST), Islamabad 44000, Pakistan

**Keywords:** Ni–Al_2_O_3_ nanocomposite coatings, wear, reciprocating motion, environmental friendly refrigerant, low carbon technology

## Abstract

This article presents a wear study of Ni–Al_2_O_3_ nanocomposite coatings in comparison to uncoated steel contacts under reciprocating motion. A ball-on-flat type contact configuration has been used in this study in which a reciprocating flat steel sample has been used in a coated and uncoated state against a stationary steel ball under refrigerant lubrication. The next generation of environmentally friendly refrigerant HFE-7000 has been used itself as lubricant in this study without the influence of any external lubricant. The thermodynamic applications and performance of HFE-7000 is being studied worldwide, as it is replacing the previous generation of refrigerants. No work however has been previously performed to evaluate the wear performance of HFE-7000 using nanocomposite coatings. The wear scar developed on each of the flat and ball samples was studied using a Scanning Electron Microscope (SEM). The micrographs show that a combination of adhesive and abrasive wear occurs when using uncoated steel samples. Micro-delamination is observed in the case of Ni–Al_2_O_3_ nanocomposite coatings accompanied by adhesive and abrasive wear. Wear volume of the wear track was calculated using a White Light Interferometer. Energy-Dispersive X-ray Spectroscopic (EDS) analysis of the samples reveals fluorine and oxygen on the rubbing parts when tested using coated as well as uncoated samples. The formation of these fluorinated and oxygenated tribo-films helps to reduce wear and their formation is accelerated by increasing the refrigerant temperature. Ni–Al_2_O_3_ nanocomposite coatings show good wear performance at low and high loads in comparison to uncoated contacts. At intermediate loads the coated contacts resulted in increased wear, especially at low loads. This increase in wear is associated with the delamination of the coating and the slow formation of protective surface films under these testing conditions.

## 1. Introduction

The use of refrigeration and air-conditioning systems has increased considerably with an increase in the global population. The ever increasing demand and usage of air-conditioning and cooling systems is also linked to global anthropogenic climate change. With increasing global warming, the demand on air-conditioning and refrigeration systems remains high. According to the International Energy Agency, air conditioning demands are set to grow rapidly over the coming decades [1]. An increasing number of air-conditioning and refrigeration units means increase in anthropogenic global warming, which is an inherited issue with the operation of an air-conditioning/refrigeration system. Besides rejecting heat to the atmosphere, an air-conditioning/refrigeration system also contributes towards global climate change through the type of refrigerant it employs in its thermodynamic cycle.

Artificially formulated refrigerants have been forced to change over the years, due to their damaging environmental implications [2]. Chlorofluorocarbons (CFCs) and Hydrochlorofluorocarbons (HCFCs) were the first artificially formulated refrigerants that went into production in 1930s [2]. In addition to having excellent heat transfer and thermodynamic properties CFCs and HCFCs also have phenomenal trigological properties [3,4,5,6,7,8,9,10,11,12]. The phenomenal trigological properties of CFCs and HCFCs are associated with the capability of these refrigerants to form protective surface films on the rubbing machine parts, which reduces wear and friction. However, the discovery of the catastrophic effects of CFCs and HCFCs on the Ozone layer surrounding the earth’s atmosphere in 1974 [13] led to a ban on these refrigerants by the Montreal Protocol on Substances that Deplete the Ozone Layer [14]. CFCs have higher Ozone Depletion Potential (ODP) in comparison to HCFCs. Use of CFCs was banned by the end of year 1995 in developed countries and HCFCs can no longer be used after 2020 [2].

Restrictions and bans on CFCs and HCFCs forced the introduction of another family of artificially formulated refrigerants called Hydrofluorocarbons (HFCs). HFCs had zero ODP and the thermodynamic properties of HFCs were reported to be similar to CFCs [15,16,17,18,19], making HFCs suitable substitute refrigerants. A number of studies were also conducted to evaluate the tribological behavior of HFCs in comparison to CFCs and HCFCs [3,4,5,6,7,8,9,11,12,20,21,22,23,24,25,26,27]. Almost all of the studies that compared the performance of HFCs to CFCs and HCFCs from a tribological viewpoint concluded that HFCs have inferior tribological performance in comparison to CFCs and HCFCs. The inferior tribological performance of HFCs is linked to the fact that, unlike CFCs and HCFCs, HFCs lack the capability to form protective surface films on the rubbing surfaces under normal compressor operating conditions. HFC refrigerants started getting used worldwide due to their zero ODP value and the fact that HFCs possessed good thermodynamic properties. The harmful global warming impact of HFCs were realized much later when HFCs were recognized as one of the major contributors towards global warming [28]. Kyoto Protocol to the United Nations Framework Convention of Climate Change in 1997 put restrictions on carbon dioxide and other greenhouse gases, banning HFCs from 2022 in any hermetically sealed system.

Naturally occurring refrigerants such as hydrocarbons gained interest due to the enforcement of Environmental Impact Legislation. A number of studies were performed to evaluate the tribological behavior of hydrocarbons under various operating conditions [29,30,31,32,33,34,35,36,37,38,39,40]. Hydrocarbons are considered to be more efficient thermal conductors than HFCs [2], however hydrocarbons are highly flammable, which seriously restricts their commercialization. Carbon dioxide is another naturally occurring compound that has good thermodynamic properties and has a global warming potential (GWP) value of only one. This makes carbon dioxide a good candidate to be used as a refrigerant. A number of experimental investigations have been performed to study the behavior and performance of CO_2_ [41,42,43,44,45,46,47,48,49,50,51,52,53,54]. The studies concerned with the tribological analysis of CO_2_ are mainly conducted at high pressures because of its low critical temperature. A scroll compressor was also developed for automotive air-conditioning system based on CO_2_ [55], but the operating pressure for carbon dioxide was very high which resulted in its low efficiency due to the large gas thrust. These studies show that carbon dioxide which is non-flammable and a good thermal conductor with a GWP value of only one is not a suitable replacement for HFCs, because of the requirement of very high operating pressures and special system design constraints.

Environmental Impact Legislation, the inherited safety and operational requirements concerning issues with the naturally occurring compounds, has yet again forced the introduction of the future generation of refrigerants. The future generation of refrigerants that have been introduced in the market not only have zero ODP but also have lower GWP values. HFOs (Hydrofluoroolefins) and HFEs (Hydrofluoroethers) are amongst these future generation of refrigerants. These refrigerants not only have zero ODP but also have lower GWP values compared to the previous generation of refrigerants. A number of studies have been performed by various researchers to assess the tribological behavior of HFOs [56,57,58,59,60,61] under various conditions and the studies have revealed that, unlike HFCs, Hydrofluoroolefins form protective tribo-films on the rubbing surfaces, which gives them very good tribological properties in comparison to HFCs. HFOs are, however, flammable and are termed as “mildly-flammable” which limits and restricts their application areas.

HFEs (Hydrofluoroethers) are non-flammable thermos-fluids having zero ODP and a low GWP value. HFEs are low toxic, colorless, odorless refrigerants having a number of application areas. The application areas include usage in fuel cells, in renewable solar thermal systems, in auto-cascade refrigeration systems, in high voltage transformers, in vapor degreasing applications, in chemical reactors, as lubricant carriers, as cleaning and rinsing agents, and so on [62]. Recent studies [63,64,65] have shown HFEs to have good thermal properties, particularly in renewable and green energy applications. HFE-7000 has significant industrial applications, including clean energy, low carbon technologies, aerospace and automotive applications. No work has also been performed to access the wear performance of HFE-7000 by using nanocomposite coatings. HFE-7000 has a GWP value of 530 and ODP value of zero [62], whereas HFC-134a, which is commonly used in domestic and automotive units, has zero ODP and a GWP value of 1430 [58]. Various other properties of HFE-7000 have been listed in Table 1. The purpose of this study is multifold: (a) to study the wear behavior of HFE-7000 by using steel contacts; and (b) to evaluate the wear performance and compatibility of Ni–Al_2_O_3_ nanocomposite coatings and look into the possibility to reduce wear of interacting parts of a system using HFE-7000 by using nanocomposite coatings.

Nanostructured design obtained by the dispersion of nano particles into a matrix has been demonstrated in enhancing optical, electrical, thermal and mechanical properties in comparison to conventionally used metallic materials [66,67,68]. A number of studies [69,70,71,72,73,74,75,76,77] have been performed by various different researchers to investigate the mechanical, wear, friction and corrosion performance of electrodeposited Nano Ni–Al_2_O_3_ coatings under a number of different environments and testing conditions. These studies have shown that electrodeposited Nano Ni–Al_2_O_3_ coatings can significantly improve the wear, friction and corrosion performance of rubbing parts. 

In this study the wear behavior of HFE-7000 has been studied using a modified reciprocating tribo-meter. The initial part of the study is concerned with evaluating the wear performance of HFE-7000 using two different types of steel in a ball-on-flat contact configuration. In the second part of this study the flat steel specimen was coated using the electrodeposition process, in which Nano Al_2_O_3_ particles were embedded in the nickel matrix and deposited on the flat steel substrate.

## 2. Materials and Methods

A reciprocating ball-on-flat contact configuration has been chosen for this study. The ball specimen is 10 mm in diameter made of AISI 52100 steel. The ball specimens are oil hardened, having excellent wear and deformation resistance. The balls are vacuum degassed and evenly through hardened in electric furnaces. The flat specimen is made of 230M07 (EN1A) steel and is circular in shape, having a diameter of 30 mm and thickness of 2.75 mm. AISI 52100 steel is a common material tested by researchers [35,37,42,43,45,46,50,58,61,78,79,80] when experimentally assessing the friction and wear performance of refrigerants, as it a common metal used in compressors. EN1A steel has shown good adhesion properties as a substrate for depositing Nano Ni–Al_2_O_3_ coatings using pulse current [74,75,76,77,81] and is also cheaper and easily available, in contrast to other specialized metals.

**Figure 1:**
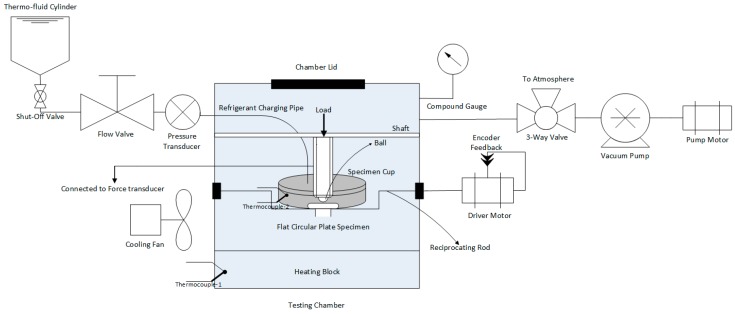
Experimental setup schematic diagram.

Phoenix Tribology reciprocating tribo-meter, TE 57 Pressurized Lubricity Tester (Phoenix Tribology, Kingsclere, UK) was modified to test the next generation of refrigerant, HFE-7000. The modified tribo-meter is schematically represented in Figure 1.

The parts that are to be evaluated are fixed in position in the testing chamber. The flat circular sample is positioned on the oscillating rod inside the cup. A wire-type thermocouple is positioned to directly read the temperature of the refrigerant in the specimen cup. Grub screws are used to fix the steel ball in the ball holder. The ball holder is then fastened to the horizontal shaft. The shaft not only holds the ball holder in position but is also used to apply the vertical normal load. Once the samples have been positioned the chamber lid is closed and a vacuum pump is used to vacuum the chamber in order to reduce the effects of ambient atmosphere and air on the testing. Once the chamber has been vacuumed, the refrigerant is introduced in the chamber. HFE-7000 has a boiling point of 34 °C and is liquid under normal atmospheric pressure and temperature. Gravity and the vacuum helps HFE-7000 to flow from the storage cylinder into the specimen cup. A sufficient amount of HFE-7000 is introduced in the chamber so that it not only fills the cups completely but also overflows and is gathered at the bottom of the testing chamber where the heating block is located. The additional overflown refrigerant helps maintain the temperature inside the chamber. The temperature of the fluid and the oscillating frequency are controlled by feedback PID (Proportional–Integral–Derivative) control. The pressure of the chamber is recorded and monitored by a pressure transducer and a pressure gauge. All the data is monitored, recorded and controlled using specialized software, COMPEND. The behavior of the refrigerant was studied under relatively low loads and temperatures because of its wide application areas and due to the fact that it is not only used in compressors.

## 3. Experimentation

The experimentation section is divided into two main parts. The first part of the experimentation is concerned with the wear study of rubbing steel parts in the presence of HFE-7000 under fully lubricated conditions. The second part of the study explains the development of Nano Ni–Al_2_O_3_ coatings using the pulse current technique, which is followed by the wear study of the coated samples under identical testing conditions as the uncoated samples.

### 3.1. Sample Preparation

Sample preparation involves preparing the surfaces of the flat circular steel specimens to the desired conditions for testing. Sample preparation for uncoated samples is discussed in Section 3.1.1 and the sample preparation of coated samples is discussed in Section 3.1.2 respectively.

#### 3.1.1. Sample Preparation of Uncoated Samples

The flat circular specimens were machined into a thickness of 2.75 mm and a diameter of 30 mm using conventional machining methods. Each sample was then grinded and polished to reach an average surface roughness value of 0.1 μm. Each sample was then conditioned with acetone (Fisher Scientific, Pittsburgh, PA, USA) for 5 min in an ultrasonic bath (Grant Instruments, Shepreth, UK) after which it was dried using a specimen drier and warm air. The grinding, polishing and surface conditioning of the samples with acetone also insured the removal of any oxide or unwanted surface films from the metal surface. 

#### 3.1.2. Sample Preparation of Coated Samples

Nano Ni–Al_2_O_3_ coatings were prepared using the pulse electrodeposition process with a coating thickness of ~10 μm on the EN1A steel substrate. The electrolyte was prepared using NiCl_2_·6H_2_O (48 g/L), NiSO_4_·6H_2_O (265 g/L), H_3_BO_3_ (31 g/L), Nano Al_2_O_3_ (20 g/L). The size of the Nano Al_2_O_3_ particles was less than 50 nm, as given by the manufacturer (Sigma-Aldrich, Gillingham, UK). The solution was magnetically stirred for 24 h, after which it was ultrasonically agitated for an additional 4 h. This ensured the proper suspension and dispersion of the particles inside the solution. The next step involved heating the solution to 40 °C. After being heated to 40 °C, the solution is ready to be used. Each machined flat circular steel specimen was grinded and polished to achieve an average surface roughness of 0.05 μm, after which it was conditioned with acetone in an ultrasonic bath for 5 min. Once the EN1A steel sample had been prepared it was suspended in the prepared solution and was used as cathode. A pure nickel sheet was also suspended in the solution and was used as the anode. The electrodeposition was started once everything was ready and in place. The pulse parameters were kept constant during the deposition process. The current density was set at (3 A/dm^2^), the duty cycle was kept at 20% and the pulse on-off time was maintained at (20 ms–80 ms). During ON-time current is applied and during OFF-time current is kept at zero. The solution was constantly magnetically stirred, ultrasonically agitated and kept at a temperature of 40 °C during the complete coating process. The coating process was stopped after 1 h i.e. (T_ON_ + T_OFF_ = 3600 s). 

**Figure 2:**
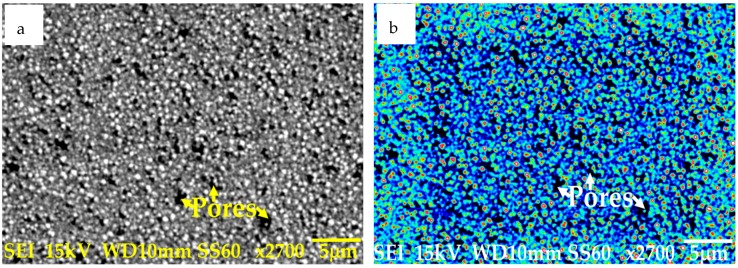
Micrograph of Ni–Al_2_O_3_ nanocomposite coating (**a**) High magnification image (**b**) False color applied to the high magnification image.

The microstructure of the prepared coating was studied using a Scanning Electron Microscope (JEOL, Tokyo, Japan). High magnification images of the prepared nanocomposite coating are presented in Figure 2. The microstructure of the prepared Ni–Al_2_O_3_ nanocomposite coating is similar to the microstructure reported by various other researchers [74,75,76,81]. The high magnification image shown in Figure 2a shows that there are pores on the surface of the electrodeposited nanocomposite coating. These pores are a characteristic of Ni–Al_2_O_3_ nanocomposite coatings [76]. Figure 2b shows the nanocomposite coating after the application of false color to the Scanning Electron Microscope (SEM) image. The colored image better reveals the pores on the coating, which are randomly distributed and are visible as dark colored spots in the colored image. 

**Figure 3:**
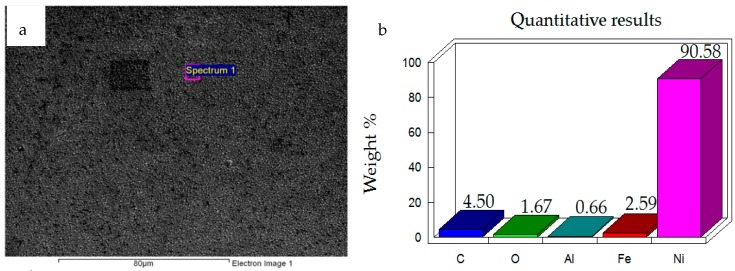
(**a**) Micrograph of Ni–Al_2_O_3_ nanocomposite coating (**b**) Energy-Dispersive X-ray Spectroscope (EDS) analysis results.

Energy-Dispersive X-ray Spectroscopic (EDS, JEOL, Tokyo, Japan) Analysis was performed at various regions on the surface of the nanocomposite coating. One of the results obtained by performing EDS analysis is shown in Figure 3b, which clearly shows the presence of Nickel, Aluminum and Oxygen confirming the deposition of Nickel on the steel substrate with Nano Al_2_O_3_ particles embedded in the Nickel matrix.

The pre-test elemental analysis and surface examination of the prepared coatings show that the Ni–Al_2_O_3_ nanocomposite coatings have been successfully prepared.

### 3.2. Testing

Both the uncoated and coated sample were tested against 52100 steel balls. Testing was performed using two different temperatures, 20 °C and 40 °C with three different loads 10 N, 20 N and 30 N. The stroke length was kept constant at 5 mm for all the tests. All the experiments were conducted at a constant frequency of 5 Hz. Each experiment was performed at least twice to ensure repeatability. A PID-controlled feedback algorithm was used to maintain the temperature during the course of a test. Before starting a test, the refrigerant was heated to the desired temperature and the temperature was maintained for 1 h. After maintaining the temperature of the refrigerant for 1 h, a test was started. Each test lasted for 2 h. The mechanical properties of all the samples were measured and are summarized in Table 2. It can be seen from the values in Table 2 that the mechanical surface properties of steel are significantly improved with the application of Ni–Al_2_O_3_ nanocomposite coating. The hardness improves from 180 HV to 450 HV and Elastic Modulus improves from 200 GPa to 280 GPa. The elastic modulus and hardness of the coated samples were measured using a Nano-indentation tester (Micro Materials, Wrexham, UK). The hardness of the steel ball and the uncoated steel specimen were measured using a Vickers hardness tester (BUEHLER, Lake Bluff, IL, USA). The average surface roughness of all the samples was measured using the white light interferometer (ZYGO, Zygo Corporation, Middlefield, CT, USA). 

## 4. Results and Discussion 

This section has been divided into further subsections to clearly state the findings of all the coated and uncoated tests. Section 4.1 covers the study of the uncoated flat circular specimens while the results of the Nano Ni–Al_2_O_3_ coated specimens are presented in Section 4.2. A brief explanation of the observations of each test is given as it is presented. Full explanation of all the observations is given at the end of Section 4.

Each sample pair, that is, the flat circular EN1A steel sample and the 52100 steel ball, were analyzed. Post-test analyses was performed on each sample pair initially under a Scanning Electron Microscope (SEM) to observe the wear mechanism at the micron level. Energy-Dispersive X-ray Spectroscopic (EDS) Analysis was done on each sample at various regions of the wear scar for the elemental analysis and chemical characterization. The wear scar on each of the flat circular specimens was stitched using a white light interferometer (ZYGO). The stitched wear scar presented the wear profile, which was used to calculate the wear volume.

### 4.1. Wear of Uncoated Specimens

**Figure 4:**
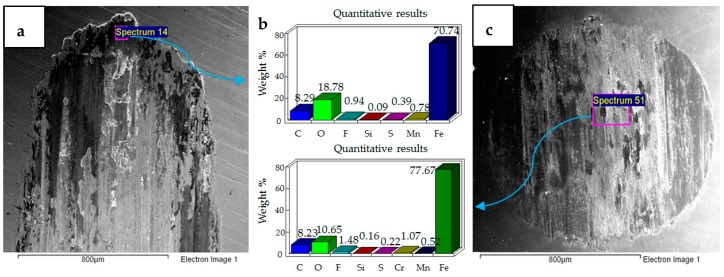
Uncoated samples. Operating conditions 10 N, 20 °C: (**a**) Scanning Electron Microscope (SEM) image of flat specimen (**b**) EDS analysis results (**c**) SEM image of ball.

**Figure 5:**
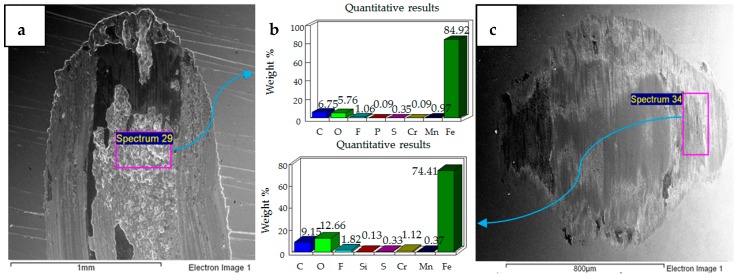
Uncoated samples. Operating conditions 20 N, 20 °C: (**a**) SEM image of flat specimen (**b**) EDS analysis results (**c**) SEM image of ball.

**Figure 6:**
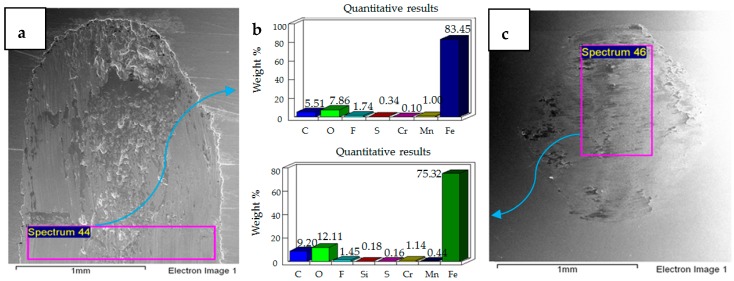
Uncoated samples. Operating conditions 30 N, 20 °C: (**a**) SEM image of flat specimen (**b**) EDS analysis results (**c**) SEM image of ball.

**Figure 7:**
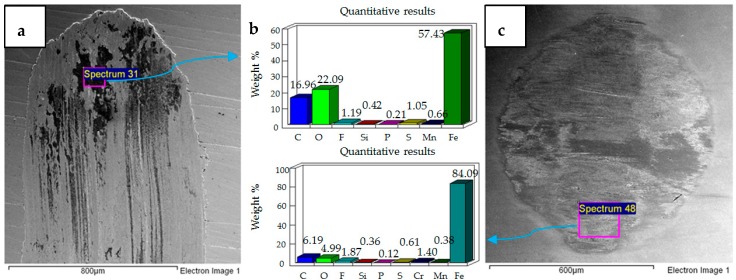
Uncoated samples. Operating conditions 10 N, 40 °C: (**a**) SEM image of flat specimen (**b**) EDS analysis results (**c**) SEM image of ball.

**Figure 8:**
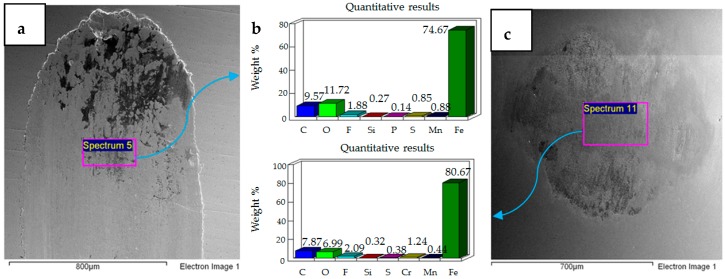
Uncoated samples. Operating conditions 20 N, 40 °C: (**a**) SEM image of flat specimen (**b**) EDS analysis results (**c**) SEM image of ball.

**Figure 9:**
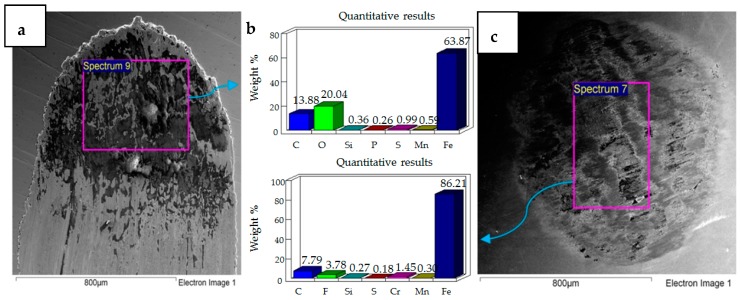
Uncoated samples. Operating conditions 30 N, 40 °C: (**a**) SEM image of flat specimen (**b**) EDS analysis results (**c**) SEM image of ball.

The hard 52100 steel ball ploughed through the EN1A steel, and material pileup was observed at the extreme ends of the wear tracks of the uncoated specimens. A combination of adhesive and abrasive wear was observed. Abrasive wear dominated at the mid-portions of the circular specimens. Adhesive wear was more prominent at the extreme ends and boundaries of the wear track. Under the application of normal load and reciprocating motion, the soft EN1A steel asperity junctions with the steel ball break and become adhered to the hard steel ball. The micrographs and EDS analysis results of all the tests are presented in Figure 4, Figure 5, Figure 6, Figure 7, Figure 8 and Figure 9.

The results of the test performed at 10 N, 20 °C are presented in Figure 4. SEM micrographs of the flat specimen reveal wear as a combination of adhesive and abrasive wear. The SEM image of the steel ball indicates scratch marks, along with a strong presence of adhered particles from the flat EN1A steel specimen, which implies that adhesive wear is more dominant under these operating conditions. The EDS analysis on the flat circular specimen, as well as on the steel ball, reveal a presence of fluorine and oxygen, which indicates the formation of tribo-films. EDS analysis revealed similar results at the mid-section of the wear track, as well as at the other extreme end of the wear scar.

SEM micrographs of 20 N, 20 °C are presented in Figure 5. Compared to 10 N/20 °C, 20 N/20 °C operating conditions produced more abrasive wear of both the flat and ball samples. EDS analysis also revealed the transfer of particles from the ball onto the flat circular specimen, along with the presence of oxygen and fluorine on the rubbing surfaces. 

SEM micrographs of 30 N, 20 °C are shown in Figure 6. Under these operating conditions the wear mechanism has shifted towards a combination of adhesive and abrasive wear, indicating a considerable increase in wear compared to the previous two conditions. Compared to 20 N/20 °C, which produced more abrasive wear, adhesion of EN1A steel on the hard steel ball along with scratch marks can be clearly observed at 30 N/20 °C. EDS analysis reveal the presence of oxygen and fluorine on both the specimens. Transfer of steel ball particles onto the flat specimen is also evident from the EDS results.

Results of increasing the refrigerant temperature from 20 °C to 40 °C are presented in Figure 7, Figure 8 and Figure 9. Figure 7 shows the results of 10 N/40 °C. Severe wear as observed at 10 N/20 °C is not visible at 10 N/40 °C. Unlike the wear scar at 10 N/20 °C the flat specimen at 10 N/40 °C also showed less adhesive wear, which is also evident by observing the ball specimen. EDS analysis of the ball and flat sample reveal the presence of fluorine and oxygen under these operating conditions as well.

Figure 8 shows the micrographs and the results of EDS analysis of operating conditions of 20 N, 40 °C. Similar to 20 N/20 °C, wear at operating conditions of 20 N/40 °C is also mainly due to abrasion. However, unlike 20 N/20 °C, SEM images show that less wear is generated at 20 N/40 °C. Fluorine and oxygen were also detected on the ball and flat sample at these operating conditions. 

Figure 9 shows the micrographs and EDS results of 30 N, 40 °C testing conditions. The micrographs show a combination of adhesive and abrasive wear similar to the test performed at 30 N, 20 °C. EDS analysis of the wear scar on the flat sample shows a strong presence of oxygen but no fluorine. EDS results of the ball sample, on the other hand, show fluorine on the top surface but no oxygen. These EDS results are different from all the other tests of the uncoated samples, as fluorine and oxygen were both detected under each of the other testing conditions on the ball, as well as the flat specimen.

**Figure 10:**
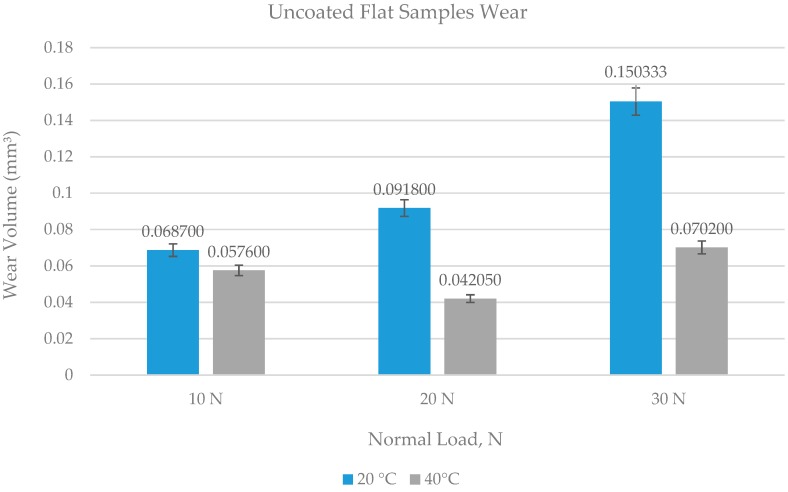
Wear volume of uncoated flat specimens.

The wear scar on each of the flat circular specimens was stitched using the white light interferometer (ZYGO). The stitched image provided a complete 3D profile of the wear scar and was used to calculate the wear volume. The type of 3D images obtained from ZYGO are shown in Figures 17 and 18 for the coated samples; similar images were obtained for the uncoated samples, as well from ZYGO. The wear volume generated at each of the operating temperatures and load for the flat uncoated samples is shown in Figure 10. Comparing the effect of the increase in temperature from 20 °C to 40 °C at any given load shows a decrease in wear volume. The effect of increasing the refrigerant temperature on wear becomes more prominent with increasing load. Increasing the refrigerant temperature from 20 °C to 40 °C reduces wear by more than 50% at 20 N and 30 N. This reduction in wear is due to the formation of protective tribo-films on the top surface of the rubbing parts. 

HFE-7000 disassociates with the application of load and constant reciprocating mechanical motion, which leads to the formation of new compounds on the freshly exposed surfaces. These fluorinated and oxygenated compounds protect the top surface. Increase in temperature from 20 °C to 40 °C at any given load decreases the viscosity of the refrigerant, reducing the separation between the rubbing parts, which should result in increased wear due to more asperity interactions. However, increase in temperature has shown a decrease in wear volume at any given load. This indicates that an increase in temperature at the same applied load increases the reactivity of HFE-7000 with the rubbing metals, resulting in a faster formation of protective tribo-films.

### 4.2. Wear of Coated Specimens

**Figure 11:**
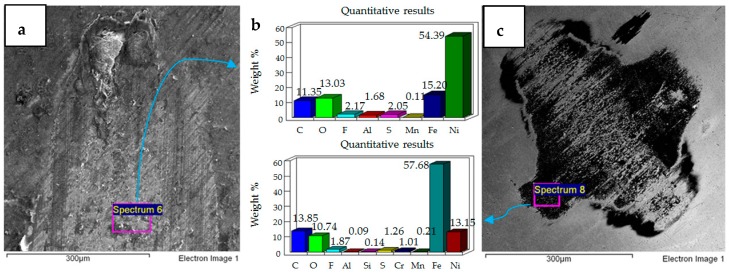
Ni–Al_2_O_3_ coated flat specimen. Operating conditions 10 N, 20 °C: (**a**) SEM image of flat specimen (**b**) EDS analysis results (**c**) SEM image of ball.

**Figure 12:**
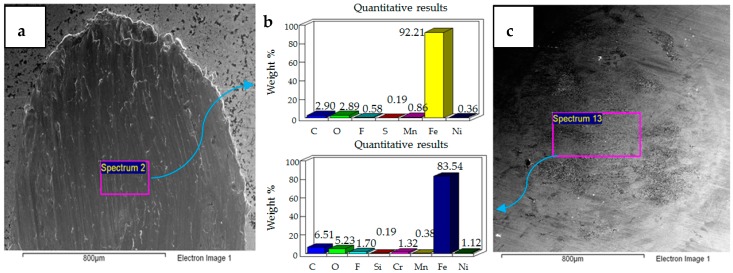
Ni–Al_2_O_3_ coated flat specimen. Operating conditions 20 N, 20 °C: (**a**) SEM image of flat specimen (**b**) EDS analysis results (**c**) SEM image of ball.

**Figure 13:**
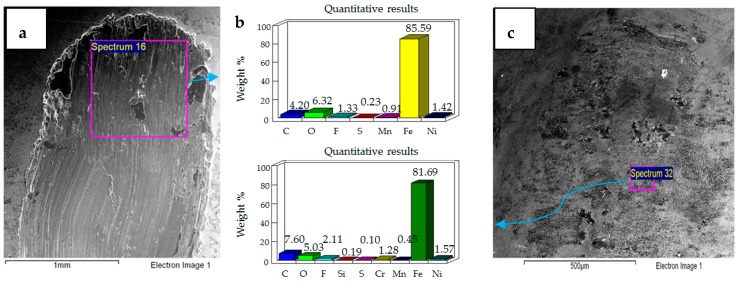
Ni–Al_2_O_3_ coated flat specimen. Operating conditions 30 N, 20 °C: (**a**) SEM image of flat specimen (**b**) EDS analysis results (**c**) SEM image of ball.

**Figure 14:**
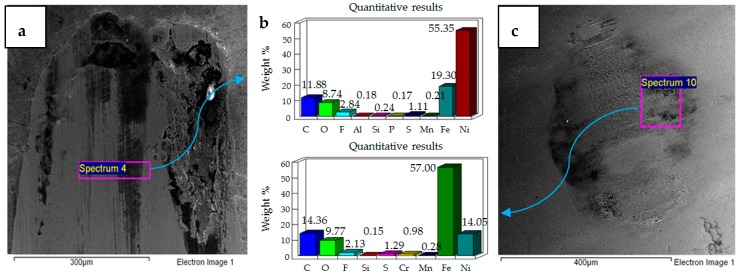
Ni–Al_2_O_3_ coated flat specimen. Operating conditions 10 N, 40 °C: (**a**) SEM image of flat specimen (**b**) EDS analysis results (**c**) SEM image of ball.

**Figure 15:**
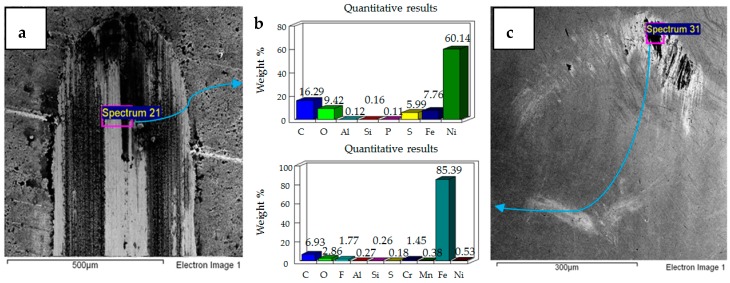
Ni–Al_2_O_3_ coated flat specimen. Operating conditions 20 N, 40 °C: (**a**) SEM image of flat specimen (**b**) EDS analysis results (**c**) SEM image of ball.

**Figure 16:**
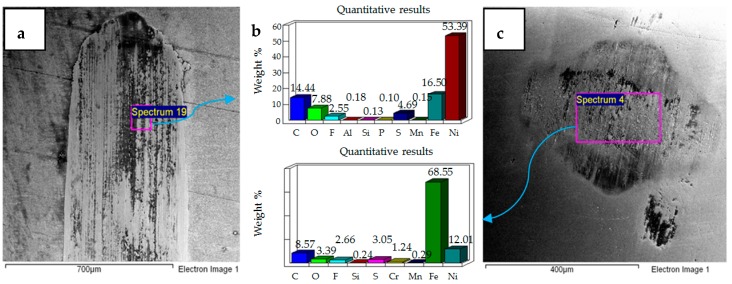
Ni–Al_2_O_3_ coated flat specimen. Operating conditions 30 N, 40 °C: (**a**) SEM image of flat specimen (**b**) EDS analysis results (**c**) SEM image of ball.

Ni–Al_2_O_3_ nanocomposite coatings were tested under identical operating conditions as the uncoated samples. EDS analysis was performed on each of the samples tested and the wear on all the samples was examined using SEM. Micrographs and EDS results of the coated samples have been presented in Figure 11, Figure 12, Figure 13, Figure 14, Figure 15 and Figure 16.

Micrographs and EDS results of flat Ni–Al_2_O_3_ nanocoated samples against 52100 steel ball at 10 N, 20 °C are shown in Figure 11. Figure 11a shows the micrograph of wear on the flat specimen. The image shows micro-delamination of the coating and exposure of the steel substrate. Ni–Al_2_O_3_ nanocomposite coatings have been reported to delaminate under reciprocating motion [76]. Micro-delamination of the coating was observed throughout the wear scar. The coating under these operating conditions is, however, still very much intact, and comparing the coated steel sample to the uncoated steel sample at 10 N/20 °C shows a reduction in wear. Micrographs of the steel ball show a combination of adhesive and abrasive wear, along with the adhesion of the delimited particles on the steel ball. Post-test EDS analysis of the steel ball shows a presence of nickel and aluminum, indicating adhesion of the delaminated coating from the flat sample on the steel ball along with fluorine and oxygen. EDS analysis on the flat sample shows iron, indicating the exposure of the steel substrate. Fluorine and oxygen were also detected on the flat sample, implying the formation of tribo-films.

The effect of increasing the load from 10 N to 20 N for the coated sample at a refrigerant temperature of 20 °C is shown in Figure 12. Figure 12a shows the micrographs of the flat specimen. This image is very different from the image obtained at 10 N/20 °C. EDS analysis of the flat specimen reveals a very strong percentage of iron, indicating that the applied coating has not only been delaminated but has also been completely worn out throughout the wear track. EDS results of the wear scar show only traces of nickel and a slight presence of oxygen and fluorine. Comparing the EDS results of the flat coated sample to the flat uncoated sample under the same testing conditions shows a lesser presence of oxygen and fluorine, which implies that protective tribo-films are not well-formed in the case of the coated sample under these testing conditions. The micrograph of the ball sample shows a combination of abrasive and adhesive wear, with abrasive wear being the more dominant wear mechanism. In comparison to the flat steel sample, the EDS results of the ball sample show a stronger presence of oxygen and fluorine, indicating that under these testing conditions the tribo-films are being more easily formed on the ball sample.

The results of increasing the normal load further from 20 N to 30 N at 20 °C refrigerant temperature are shown in Figure 13. The micrograph and EDS analysis of the flat sample show that the coating has been completely delaminated and worn out under these operating conditions as well. Elemental analysis shows the presence of fluorine and oxygen on the flat as well as ball samples. Increasing load from 20 N to 30 N at 20 °C shows an increase in adhesive wear, which is evident from the flat and ball samples indicating an increase in wear volume. The wear mechanism of the uncoated sample at 30 N/20 °C is similar to the coated sample at 30 N/20 °C, in both of these cases wear is a combination of adhesive and abrasive wear. However there is more adhesive wear for the coated sample because of the adhesion of the delaminated coating on the ball.

The effect of increasing the refrigerant from 20 °C to 40 °C at 10 N load is shown in Figure 14. The micrograph of the flat sample shows more delamination of the coating at the sides and top of the wear scar. This micrograph is different from 20 °C/10 N, in which delamination of the coating was observed throughout the wear track. EDS analysis of the wear scar shows the presence of iron, along with oxygen and fluorine, indicating formation of surface films and micro-delamination of the coating along the wear track. The micrograph of the ball specimen at these operating conditions shows less adhesive wear in comparison to the micrograph of the ball sample at 20 °C/10 N. EDS analysis of the ball sample also shows the presence of oxygen and fluorine. Wear of the coated sample at 40 °C/10 N presents a combination of adhesive and abrasive wear whereas the uncoated sample at 40 °C/10 N shows more abrasive wear.

Increasing load from 10 N to 20 N at refrigerant temperature 40 °C increases abrasive wear as shown in Figure 15. The micrograph and EDS results show micro-delamination of the coating along the sides of the wear track due to abrasion. Unlike 10 N/40 °C, which displayed strong delamination at the top and extreme corners of the wear track, at 20 N/40 °C the wear track shows a more uniform micro-delamination of the coating along the wear scar. Similar to 20 N/ 20 °C, abrasive wear is the more dominant wear phenomenon at 20 N/ 40 °C as well. However, unlike 20 N/ 20 °C, at which a total delamination of the coating was observed, the coating at 20 N/ 40 °C is still very much intact, indicating a reduction in wear. Coated and uncoated samples at 20 N/ 40 °C present similar results in terms of wear mechanism, as abrasive wear was dominant in the case of coated as well as uncoated samples under these testing conditions. 

Similar to 30 N/20 °C testing conditions, increasing the load from 20 N to 30 N at 40 °C results in a combination of adhesive and abrasive wear, also shown in Figure 16. However, unlike 30 N/20 °C, at which a complete delamination of the coating was observed, the coating at 30 N/40 °C is still intact. The EDS analysis of both the flat and ball samples reveal the presence of fluorine and oxygen. EDS results and micrograph of the flat sample show more micro-delamination of the coating along the center of the wear scar as compared to the sides. The micrograph of the coated flat steel sample also demonstrates a combination of adhesive and abrasive wear similar to the uncoated sample tested at 30 N/40 °C, but differs in regards to the extent of wear. The coated flat sample shows less wear damage in comparison to the uncoated sample. The steel ball tested with the coated sample also shows less wear than the ball used in the uncoated test.

**Figure 17:**
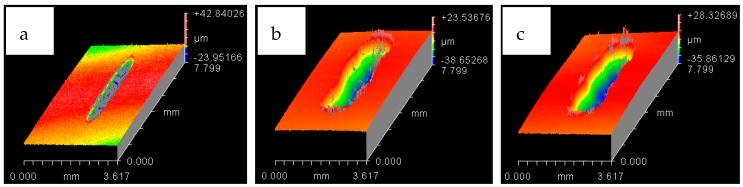
Wear track for HFE-7000 temperature 20 °C with Ni–Al_2_O_3_ coated specimens. At normal loads of: (**a**) 10 N (**b**) 20 N (**c**) 30 N.

**Figure 18:**
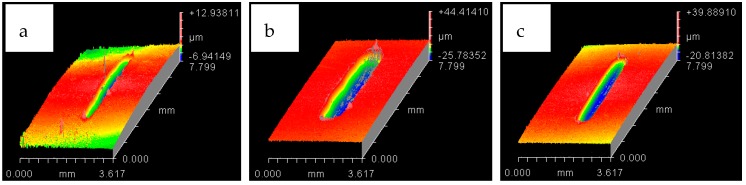
Wear track for HFE-7000 temperature 40 °C with Ni–Al_2_O_3_ coated specimens. At normal loads of: (**a**) 10 N (**b**) 20 N (**c**) 30 N.

The white light interferometer (ZYGO) was also used to stitch the wear track of the flat coated samples to provide the complete 3D plot of the wear scar. The 3D images of the wear track for both the testing temperatures have been presented in Figure 17 and Figure 18. The stitched 3D profiles of the wear tracks were used to calculate the wear volume for each of the testing conditions.

**Figure 19:**
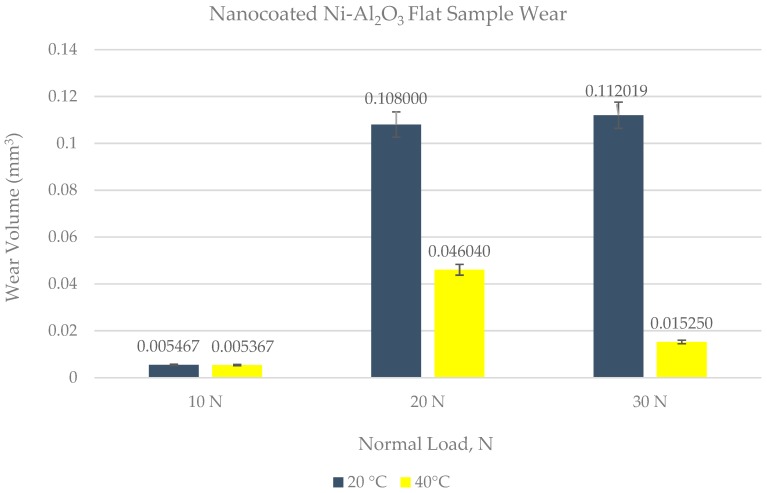
Wear volume of Ni–Al_2_O_3_ coated flat specimens.

Wear volume of each of the coated flat samples was determined using the 3D images shown in Figure 17 and Figure 18 generated by ZYGO. The results of the wear volume of the coated flat samples have been presented in Figure 19. Similar to the uncoated samples, increase in load at the same temperature increases wear. Less wear is generated at 10 N/20 °C by using a coated flat specimen in comparison to uncoated flat sample at the sample testing conditions. This indicates that at low loads and lower HFE-7000 temperature, Ni–Al_2_O_3_ nanocomposite coatings help reduce wear. Wear is influenced by the surface mechanical properties which improve considerably by the application of Ni–Al_2_O_3_ nanocomposite coatings which results in wear reduction. The hardness, elastic modulus and the surface roughness are all improved by the application of Ni–Al_2_O_3_ nanocomposite coatings in comparison to the uncoated samples. The smoother surface of the coated samples have an average surface roughness value of only 0.045 μm, whereas the uncoated flat specimens have a higher average surface roughness value of 0.1 μm. Smoother surface of the coated samples means lesser asperity interactions, resulting in reduction in wear. Another reason for reduction in wear volume when using coated specimens is due to the micro-pores that exist in Ni–Al_2_O_3_ nanocomposite coatings, as shown in Figure 2; these pores help retain the liquid refrigerant, increasing the lubricity of the rubbing parts. Micro dimpled grooves and cavities in the surface have been reported to improve the tribological performance of interacting parts [82,83]. The presence of micro-pores in this case similarly helped improve the wear performance.

For 20 °C refrigerant temperature, wear volume increases rapidly by increasing the load from 10 N to 20 N for coated flat specimens. In the case of uncoated specimens, an increase in load from 10 N to 20 N does result in slightly higher wear, but the rate of increase in wear volume is not as sharp in the case of an uncoated specimen. Also comparing the wear volume of the uncoated specimen to the coated specimen at 20 N/20 °C shows that more wear has occurred when using the coated specimen. This shows that increasing the load at a low refrigerant temperature of 20 °C has an adverse effect on wear. The micrographs of the wear scar as presented in Figure 12 show a total delamination of the coating. The EDS results of the wear scar show scarce presence of oxygen and fluorine. This shows that not only has the coating completely worn out under these conditions, the protective surface films have also not fully formed on the surface of the flat specimen. This result shows that using this coating at low temperatures and slightly higher loads results in an increase in wear. EDS results of the ball show the presence of oxygen and fluorine, indicating that protective surface films are being formed on the counter-face under these operating conditions. 

A further increase in load at 20 °C from 20 N to 30 N further increases wear. The wear volume generated using coatings is less compared to the uncoated sample under these conditions. The coating has also been completely worn out at 30 N/20 °C, similar to at 20 N/20 °C. Similar to the uncoated test at 30 N/20 °C the coated test at 30 N/20 °C also produced a combination of adhesive and abrasive wear. Adhesive wear is more prominent in the coating study because of the delamination and wear of the coating, which becomes adhered to the steel ball under constant reciprocating motion. The normal applied load has been further increased by 10 N, but the wear volume reduces by using coating unlike the previous testing conditions, that is, 20 N/20 °C. EDS results of the ball and flat sample show a higher percentage of fluorine and oxygen in comparison to the 20 N/20 °C testing conditions. This shows that increasing the load at a constant temperature helps the faster chemical breakdown of HFE-7000, which results in the accelerated formation of protective surface films which help reduce wear.

Doubling the refrigerant temperature from 20 °C to 40 °C at 10 N load results in the reduction of wear compared to the uncoated test at 10 N/40 °C. Doubling the temperature also shows a decrease in wear volume compared to the 10 N/20 °C coated test. The delamination process of the coating is slow under these testing conditions, which is mainly due to the low load and healthy formation of oxygenated/fluorinated tribo-films on the top surfaces of the rubbing parts at elevated temperatures. 

Increasing the load to 20 N at 40 °C increases wear of the coated flat sample, which is different from the results obtained when using uncoated parts. The uncoated flat specimen showed the least amount of wear at 20 N/40 °C amongst all the uncoated tests, showing an optimum load and temperature combination. The micrographs and EDS analysis of the wear track of the coated flat sample show that there is micro-delamination of the coating and the presence of oxygen and fluorine. Comparing the wear volumes of the coated and uncoated wear tracks shows that the difference in the wear volumes is not as high as it was at 20 °C at the same load. A reason for this increased wear when using the coating is the micro-delamination of the coating, which increases the wear volume. Comparing the results of the coated contacts at 20 °C and 40 °C at 20 N load shows that increasing the refrigerant temperature has a high positive impact on reducing wear. 

Increasing the load further to 30 N at 40 °C reduces wear. This wear volume is less than the wear generated by using uncoated samples. This wear is also less in comparison to the wear at 30 N/20 °C. The EDS results and micrographs show that, although there is micro-delamination of the coating, the coating is still well adhered to the steel substrate. This micro-delamination is also less than the micro-delamination at 20 N/40 °C. These results show that increasing the temperature increases the reactivity of the refrigerant with the rubbing parts, resulting in the accelerated formation of protective tribo-films which reduce wear. 

**Figure 20:**
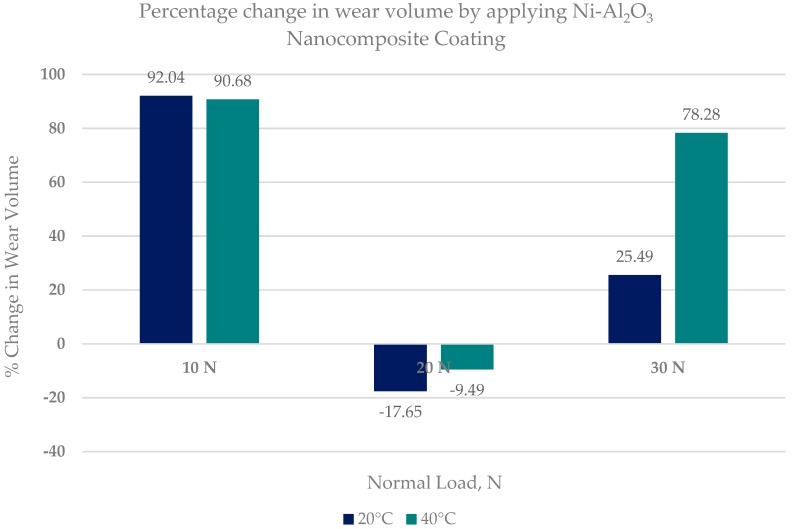
Percentage change in the wear volume of flat specimens by using Ni–Al_2_O_3_ nanocomposite coatings.

The least amount of wear for coated specimens was observed at a low load of 10 N and at a high refrigerant temperature of 40 °C. The percentage change in wear volume by using Ni–Al_2_O_3_ nanocomposite coatings in comparison uncoated samples is shown in Figure 20.

Figure 20 shows that applying Ni–Al_2_O_3_ nanocomposite coatings has a positive effect on wear and these coatings can reduce wear by more than 90% at low loads. Increasing the load to 20 N has an adverse effect on wear of coated flat samples especially at low refrigerant temperature. However increasing the applied load further to 30 N reduces wear as well in comparison to the uncoated tests. This shows that Ni–Al_2_O_3_ nanocomposite coatings can significantly reduce wear in systems employing HFE-7000 at low and high operating loads. 

## 5. Conclusions

In this work, wear performance analysis of Ni–Al_2_O_3_ nanocomposite coating was performed under nonconventional, that is, refrigerant lubrication and the results were compared to uncoated steel samples. The study concludes the following points:

- A modified tribo-meter was used to study the wear performance of Ni–Al_2_O_3_ nanocomposite coating under HFE-7000 refrigerant lubrication under varying operating conditions.
- Ni–Al_2_O_3_ nanocomposite coatings were successfully developed using the pulse coating technique. The wear mechanism of the coatings developed has been studied and has been compared to uncoated contacts under the same operating conditions.
- Ni–Al_2_O_3_ nanocomposite coating shows good wear performance at low loads and can reduce wear by more than 90% compared to uncoated parts. At intermediate loads, the wear performance of these coatings showed adverse effects and increased the wear by 18% at low operating temperatures of 20 °C. Further increase in load to 30 N reduced wear by 25% at the low refrigerant temperature of 20 °C and by 78% at the higher refrigerant temperature of 40 °C.
- Post-test elemental analysis of the surfaces revealed oxygen and fluorine on the interacting parts by using coated as well as uncoated steel samples. This indicates that HFE-7000 formed new compounds on the metallic surfaces, resulting in the formation of protective tribo-films which help reduce wear.
- Increase in temperature has a very positive impact on reducing wear of both the coated and uncoated samples. The ability of HFE-7000 to from protective tribo-films on the rubbing surfaces increases with increase in temperature, resulting in the accelerated formation of protective surface films on the rubbing metals.
- HFE-7000, which is an environmentally friendly future generation thermo-fluid, having good thermodynamic properties, has shown good wear performance as well.
- Wear can be reduced by using Ni–Al_2_O_3_ nanocomposite coating on EN1A-steel substrate instead of using specialized expensive metal alloys.

## 6. Future Work

Ni–Al_2_O_3_ nanocomposite coating has been examined in this study, and the results were compared to the uncoated parts of a specific surface finish. The future work should investigate other nanocomposite coatings as well to evaluate their performance and compatibility with HFE-7000. Application of Ni–Al_2_O_3_ nanocomposite coatings indicated that a considerable reduction in wear can be achieved at low and high operating loads; however, these nanocomposite coatings showed poor wear performance at intermediate loads. It would be extremely beneficial to study other nanocomposite coatings under the same operating conditions to determine which coatings perform better at intermediate loads as well. For the uncoated sample, only a single value of surface roughness has been used; it will be very useful to experimentally evaluate how uncoated EN1A steel parts of various surface finish perform under HFE-7000 refrigerant lubrication.

## Figures and Tables

**Table 1:** Various properties of hydrofluoroether (HFE)-7000 [62].

HFE-7000	
Structure	C_3_F_7_OCH_3_
Molecular Weight (g/mol)	200
Freeze Point (°C)	−122.5
Boiling Point @ 1 atmosphere (°C)	34
Critical Temperature (°C)	165
Critical Pressure (MPa)	2.48
Flash Point (°C)	None
Kinematic Viscosity @−120°C (cSt)	17
Kinematic Viscosity @20°C (cSt)	0.32
Kinematic Viscosity @40°C (cSt)	0.27
Flammability	Non-flammable
Ozone Depletion Potential (ODP)	Zero
Global Warming Potential (GWP)	530 *

* GWP 100-year integrated time horizon [62].

**Table 2:** Measured mechanical properties of all the samples used.

Specimen	Hardness (HV)	Elastic Modulus (GPa)	Average Surface Roughness (μm)
Steel ball	810	210	0.010
Uncoated steel specimen	180	200	0.1
Coated steel specimen	450 ± 25	280	0.045
